# Dynamic Oscillations Evoked by Subcallosal Cingulate Deep Brain Stimulation

**DOI:** 10.3389/fnins.2022.768355

**Published:** 2022-02-23

**Authors:** Vineet Tiruvadi, Ki Sueng Choi, Robert E. Gross, Robert Butera, Viktor Jirsa, Helen Mayberg

**Affiliations:** ^1^Emory School of Medicine, Emory University, Atlanta, GA, United States; ^2^Department of Biomedical Engineering, Georgia Tech and Emory University, Atlanta, GA, United States; ^3^School of Electrical and Computer Engineering, Georgia Tech, Atlanta, GA, United States; ^4^Icahn School of Medicine, Mount Sinai, New York, NY, United States; ^5^Institut de Neurosciences des Systèmes, Aix-Marseille Université, Marseille, France

**Keywords:** subcallosal, cingulate, DBS, dynamics, tractography

## Abstract

Deep brain stimulation (DBS) of subcallosal cingulate white matter (SCCwm) alleviates symptoms of depression, but its mechanistic effects on brain dynamics remain unclear. In this study we used novel intracranial recordings (LFP) in *n* = 6 depressed patients stimulated with DBS around the SCCwm target, observing a novel *dynamic oscillation* (DOs). We confirm that DOs in the LFP are of neural origin and consistently evoked within certain patients. We then characterize the frequency and dynamics of DOs, observing significant variability in DO behavior across patients. Under the hypothesis that LFP-DOs reflect network engagement, we characterize the white matter tracts associated with LFP-DO observations and report a preliminary observation of DO-like activity measured in a single patient's electroencephalography (dEEG). These results support further study of DOs as an objective signal for mechanistic study and connectomics guided DBS.

## 1. Introduction

Deep brain stimulation (DBS) of subcallosal cingulate white matter (SCCwm) has long term, sustained antidepressant effect in patients with treatment resistant depression (TRD) (Holtzheimer et al., 2012; Riva-Posse et al., 2018; Crowell et al., 2019). Although short-term randomized control trials (RCTs) studying DBS of the broad SCC region have thus far been equivocal (Holtzheimer et al., 2017), one contributing factor may be inconsistent stimulation of widespread networks traversed by SCCwm (Riva-Posse et al., 2018; Howell et al., 2019; Tsolaki et al., 2021). Objective measurements of network effects evoked by DBS in the SCC are needed to resolve the apparent contradiction in demonstrated efficacy and test tractography-based hypotheses of therapy (Choi et al., 2015; Waters et al., 2018).

Recent advances in clinical DBS hardware allowing for local field potential (LFP) recordings during active therapy enable opportunistic study in novel patient cohorts (Stanslaski et al., 2012, 2018; Starr, 2018). Studies in other disorders and DBS targets have demonstrated the utility of electrophysiology in both guiding and studying DBS as a network intervention (De Hemptinne et al., 2015; Swann et al., 2018; Muthuraman et al., 2020). Studies identifying oscillations evolving over time (Schiff et al., 2000; Wiest et al., 2020) suggest higher-order characterizations are also needed to accurately characterize brain network responses to DBS. Modern analytical approaches that rely on neural-network based identification of underlying dynamics can complement traditional frequency-domain analyzes to more directly inform mechanistic understanding of DBS effects on circuits (Champion et al., 2019). An understanding of the dynamics evoked by SCCwm-DBS will likely help clarify the wider network and dynamics that must be modulated to achieve therapeutic response.

In this report, we present a serendipitous observation of *dynamic oscillations* evoked by DBS initiation in particular anatomical structures that exhibit strong frequency components changing over the course of minutes. Our group opportunistically recorded LFP from the Activa PC+S™ device (Medtronic PLC, Minneapolis, MN) in a set of six treatment resistant depression (TRD) patients during initial testing of subcallosal cingulate white matter (SCCwm) DBS parameters, before initiation of chronic therapy. Here, we confirmed that DOs are of neural origin and characterized their properties before attempting a preliminary explanation of their mechanistic origins.

## 2. Materials and Methods

### 2.1. Regulatory, Patient, and Therapy

**Figure 1:**
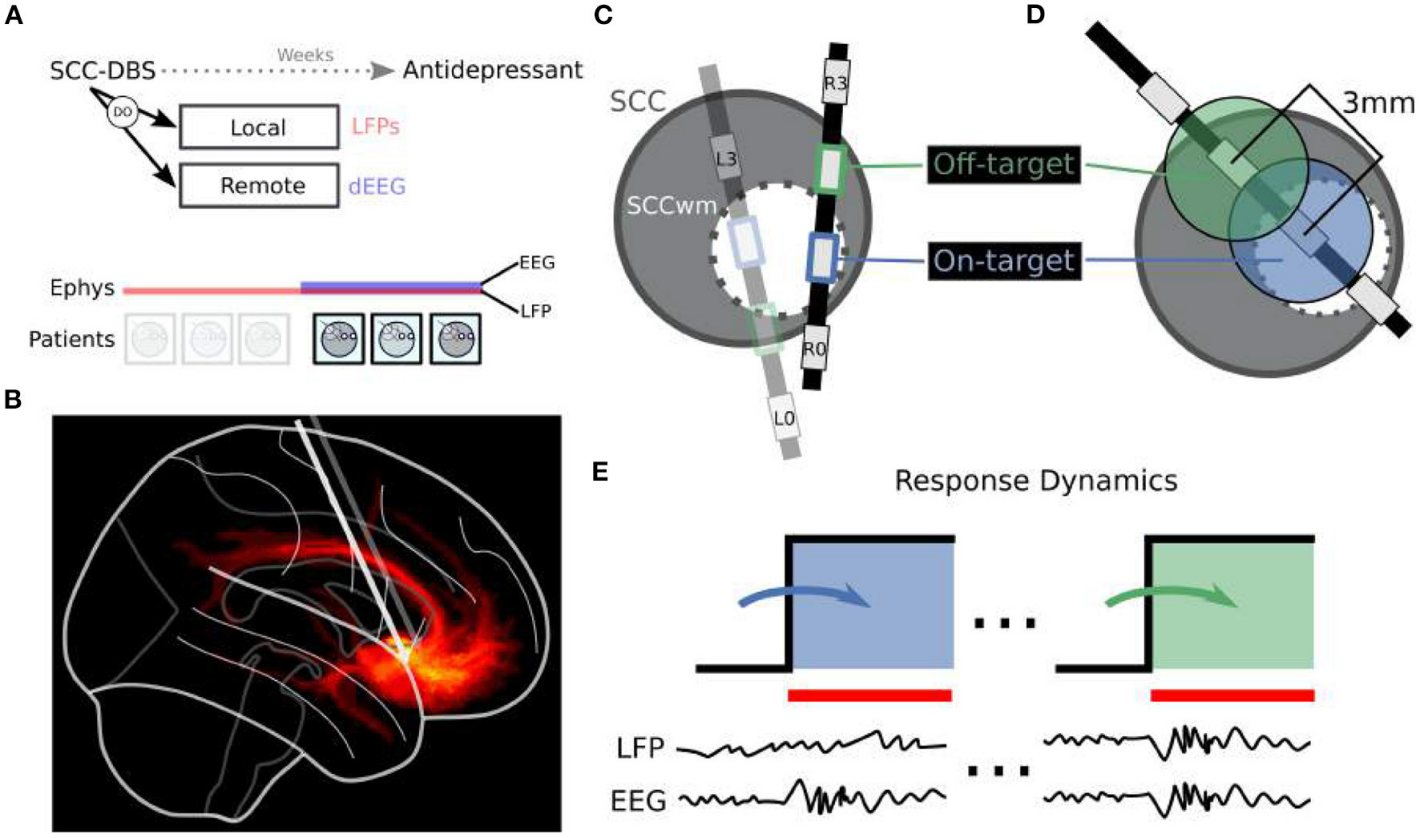
Overview of Dynamic Oscillations (DO) Identification. **(A)** Local field potentials (LFP) and dense scalp electroencephalography (dEEG) recordings are taken at therapy onset one month after implantation, with no active DBS in the interim. Six patients are included here. **(B)** DBS is targeted at patient-personalized subcallosal cingulate white matter (SCCwm). Sagittal view here demonstrates fibers being engaged at therapeutic settings. **(C)** DBS3387 leads are implanted bilaterally such that SCCwm is close to one of the center two electrodes. OnTarget electrode (blue) is the one closest to the SCCwm while OffTarget (green) is the other middle electrode. **(D)** Spacing between OnTarget and OffTarget is 1.5 mm edge-to-edge. **(E)** Separate recording sessions are performed for OnTarget and OffTarget DBS targets. LFP, with combined dEEG in *n* = 3/6 patients, were recorded continuously before and during DBS ON periods. DBS ON recordings are compared to the immediately previous DBS OFF period.

Six consecutive patients were enrolled between April 2014 and January 2016 in an IRB approved research protocol at Emory University studying the safety, efficacy, and mechanisms of SCC DBS for TRD (Figure 1A; Table 1). Written informed consent was provided by each patient to participate in the study (ClinicalTrials.gov NCT00367003, IDE G060028). Patients were recruited based on strict inclusion and exclusion criteria [described in Riva-Posse et al. (2018)], with baseline Hamilton Depression Rating Scale (HDRS-17) greater than 19. Therapeutic DBS at bilateral SCCwm was initiated one month after surgical implantation (Week C01) and maintained continuously for six months (Week C24). Experiments involving LFP±EEG were performed immediately before therapeutic DBS initiation, with the final three patients (the EEG subcohort) having simultaneous LFP and EEG on the day of therapy initiation (Figure 1A). OffTarget electrodes (Figure 1) that are not situated in the SCCwm target are stimulated only for this experimental session and not used therapeutically.

**Table 1:** Patient Information.

**Patient**	**Age at Implant**	**Sex**	**Baseline HDRS**	**End HDRS**	**OnTarget**	**OffTarget**	**EEG**
Patient 1	50	F	23	6	L2+R1	L1+R2	N
Patient 2	48	F	20	7	L2+R2	L1+R1	N
Patient 3	70	F	23	15	L2+R1	L1+R2	N
Patient 4^*^	64	M	19	6	L2+R2	L1+R1	Y
Patient 5	62	F	23	7	L1+R1	L2+R2	Y
Patient 6	57	M	20	10	L2+R1	L1+R2	Y

Six patients were included in this study. Each patient was stimulated under OnTraget and OffTarget electrodes, with simultaneous LFP and/or EEG recordings. Patient 4, with a “*,” indicates a complete LFP+EEG dataset in a patient exhibiting an LFP-DO.

### 2.2. Tractography and Implantation

Stereotactic implantation and SCCwm targeting is described in detail in Riva-Posse et al. (2018). Briefly, the SCCwm target was defined as the convergence of four white matter bundles within SCC region (forceps minor, cingulum bundle, uncinate fasciculus, and fronto-striatal fibers; Figure 1B). Diffusion tractography identified the optimal SCCwm coordinate for each patient and a center electrode on a DBS3387 lead (Medtronic PLC) was implanted at this target coordinate. Optimal contacts were confirmed with post-operative high resolution computed tomography (CT) (Riva-Posse et al., 2018), with the electrode closest to the SCCwm designated *OnTarget* and an adjacent middle-electrode labeled *OffTarget* 1.5 mm away (Figures 1C,D).

Volume of tissue activated (VTA) was simulated using StimVision in high resolution CT space for both ONTarget and OffTarget electrodes at various stimulation amplitudes 2 to 7 V and 2 to 7 A (Chaturvedi et al., 2013; Noecker et al., 2018). VTAs were then transferred to native diffusion space through high-resolution structural T1 weighted by rigid-body linear transformation and whole brain structural connectivity map was generated by probabilistic tractography in FSL toolbox (Jenkinson et al., 2012) using a individualized bilateral VTA seeds (Riva-Posse et al., 2018). Mean tractography was then calculated across all VTA using NILearn (Abraham et al., 2014), yielding an average map for OnTarget and OffTarget conditions in each patient. All voxels are then binarized into a final mask, calculated by identifying the condition that each non-zero voxel is larger in: LFP-DO+ vs. LFP-DO-.

### 2.3. Experimental Design

Multi-modal electrophysiology is collected with DBS both ON and OFF, with ON being delivered at a specific *target* in a particular *configuration*. We stimulated two *targets* in each patient: OnTarget at the patient personalized SCCwm coordinate, and OffTarget at an adjacent middle electrode whose placement is not optimized (Figures 1C,D). Each target was stimulated in one of three *configurations*: Unilateral Left DBS, Unilateral Right DBS, and Bilateral DBS (**Figures 4C,D** for examples), with 1 min washout periods between configurations. Each target required a separate 30 min recording and LFP download session.

### 2.4. Stimulation Parameters

Chronic antidepressant DBS is done with bilateral SCCwm-DBS, so only the bilateral stimulation conditions are analyzed in this report. All DBS was monopolar stimulation at therapeutic 130 Hz frequency, and supratherapeutic 6 mA amplitude with 90 μs pulsewidth, delivered to the predetermined target bilaterally. Patients were seated in a neutral position facing a blank monitor and asked to close their eyes. Before every recording session there was an EEG impedance measurement and appropriate saline application on EEG electrodes to ensure all scalp impedances were below 1 kΩ.

### 2.5. Neural Recordings

In all patients, LFP from bilateral SCC were collected and, in a subset of *n =* 3/6, dense-array EEG (dEEG) were collected. Intracranial LFP from bilateral SCC was recorded using the Activa PC+S™ device (Medtronic PLC) (Stanslaski et al., 2012). LFPs were recorded differentially around the stimulated electrode, allowing for the removal of stimulation artifact. Recordings were sampled at 422 Hz and hardware filters set at a 1 Hz high-pass filter and a 100 Hz low-pass filter.

dEEG was sampled at 1 kHz with a 100 Hz lowpass filter and a 2 Hz highpass filter. Impedances were kept below 1 kΩ throughout recordings. Recordings were measured continuously throughout a session, consisting of 1 min washout periods without active DBS and 3 min stimulation periods at a specified DBS target and configuration. Recordings are re-referenced by subtracting the average of all neighbors, then low-pass filtered at 100 Hz. The resulting continuous timeseries are analyzed with the same tools as the LFP.

### 2.6. Artifacts and Filtering

**Figure 2:**
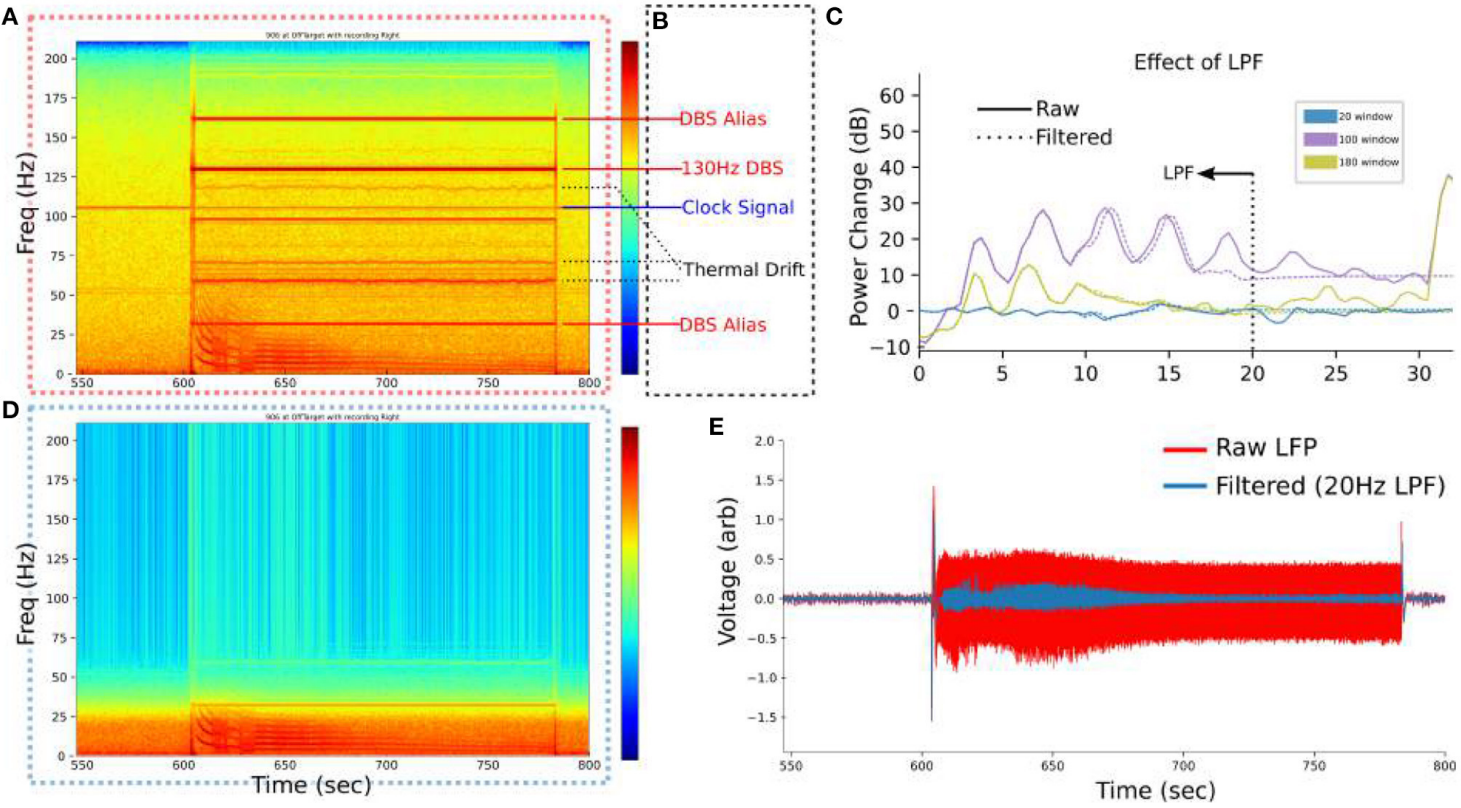
Filtering setup for SCC-LFP. **(A)** Example spectrogram of raw LFP from PC+S™. 550 to 600 s is before stimulation at 130 Hz and exhibits small artifacts at 105.5 Hz and 60 Hz. **(B)** Known artifacts in the PC+S™ become evident during stimulation. **(C)** Low pass filter at 20 Hz removes all artifacts identified in **(B)** without significantly altering the low-frequency bins where the DO manifests. **(D)** Visualization of the effect of the LPF on removing artifacts. **(E)** Time-domain signal after filtering more clearly demarcates the boundaries of the regimes.

The PC+S™ contains numerous recording artifacts that are evident in our SCC-LFP recordings (Figure 2A), attributable to known PC+S™ artifacts (Figure 2B) (Stanslaski et al., 2012; Swann et al., 2017): First, a residual 130 Hz artifact is present during DBS stimulation due to incomplete rejection of the stimulation artifact by the PC+S™ (Stanslaski et al., 2012). Second, the 130 Hz artifact has predictable harmonics due to various device processes at predictable frequencies: 32 Hz, 160 Hz being the most evident. Third, a persistent 105.5 Hz clock signal is present on the PC+S™ at all times Finally, a temperature-dependent “Thermal Drift” artifact is distinct from narrower artifacts and present above 50 Hz.

For visualization no filtering is performed to leverage device artifacts as frequency landmarks. For analyzes a low-pass 10-order Butterworth filter at 20 Hz is implemented where specified and for the SINDy analyzes. The effect of the low-pass filter is confirmed to be linear (Figures 2C,D). The low-pass filtered timeseries is used for SINDy analyzes (Figure 2E).

### 2.7. Time-Frequency Analyzes

Time-frequency (TF) representations were calculated by a Welch estimator with 1024 bins, 0% overlap, 1024 NFFT, and Blackman-Harris windowing to minimize artifact-related spectral leakage. The TF representation was visualized as a spectrogram with 50% overlap for temporal smoothing. Spectrograms were used to identify distinct time periods, or regimes, of the DO for focused analysis of time windows with similar dynamics. Analysis in the frequency domain is done by averaging across the relevant time windows in the spectrogram to yield power spectral densities (PSD). PSDs measured during active DBS are baseline corrected by subtracting the average logPSD of the final 30 s of the previous baseline period.

### 2.8. Dynamics Learning

The SINDy algorithm (Brunton et al., 2016; Champion et al., 2019), implemented in the PySINDy library (de Silva et al., 2020), was used to learn a sparse, non-linear dynamical system from recorded timeseries. SINDy uses an autoencoder architecture to build a generative model of the dynamics, or time evolution, of a set of timeseries. To apply the SINDy analysis, 20 Hz low-pass filtered timeseries are downsampled by a factor of 5 in both left and right SCC-LFP. A SINDy model is initialized with combined polynomial and Fourier basis library for both regime analysis and sliding window analysis. Sliding window analysis is done with 30 s windows and Coefficients are then clipped to −1000 to 1000 range for analysis of asymmetric influence. Regime analysis is done on non-overlapping segments of bilateral LFP-DOs, with regimes defined by visual inspection of spectrograms.

### 2.9. Code and Data

Code is available as open-source Python scripts at “https://github.com/virati/cortical_signatures.” Intermediate data can be made available upon request.

## 3. Results

### 3.1. Dynamic Oscillations (DOs) Are Evoked by DBS

**Figure 3:**
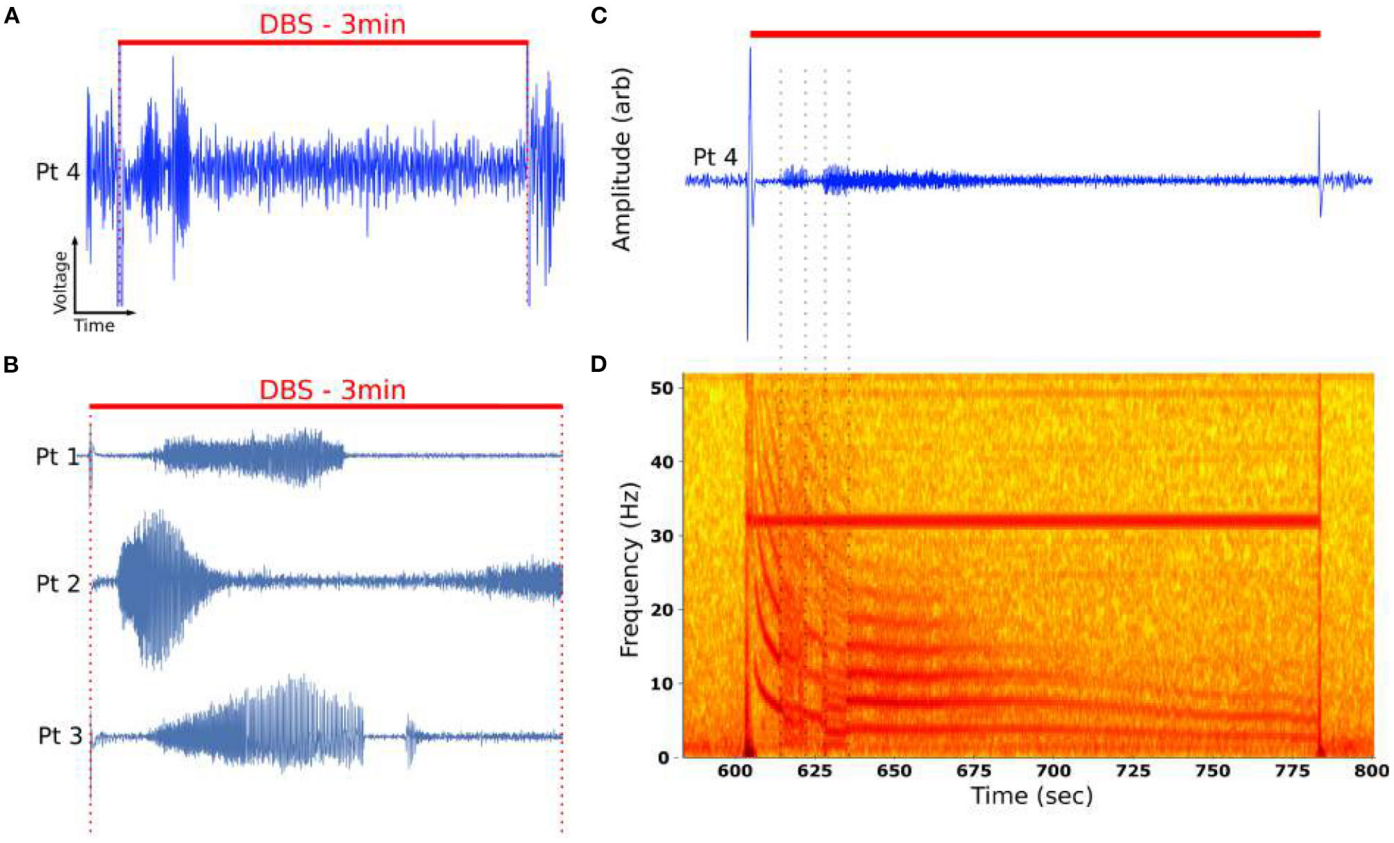
Time evolution of DOs in example DOs. **(A)** Unfiltered recording during active DBS demonstrated emergence of oscillatory activity changing over minutes of stimulation. **(B)** DOs are observed in four of six patients. **(C)** Filtered DO demonstrates distinct phases of activity marked by sharp changes in the oscillatory activity. **(D)** Characteristic spectrogram of DO from **(A)**. Spectrogram demonstrated a fundamental oscillation around 12 Hz with numerous harmonics. The fundamental changes over the course of 3 min of stimulation, mostly smoothly but punctuated by distinct transitions that are burst-like (dotted lines). The periods bounded by these distinct transitions are called *regimes*.

DOs were observed in four of six patients under bilateral DBS using the PC+S™ (Figures 3A,B), though significant variability in the dynamics were evident across all six patients (**Figure 5**).

Low-pass filtering demonstrated the presence of burst-like activity in distinct time periods, or *regimes* (Figure 3C). In the time-frequency domain, visualized by the spectrogram (Figure 3D), these oscillations have fundamental frequencies between 1 and 20 Hz and exhibit integer harmonic components, ranging from 2 to 7 components throughout the stimulation period. Several distinct regimes could be identified by their abrupt transitions (Figure 3D, dotted lines). Several regimes demonstrated continuous decay in oscillatory frequencies, with fundamental between 20 and 2 Hz (Figures 3C,D).

### 3.2. DOs Are Reproducible and Robust to Recording Hardware

**Figure 4:**
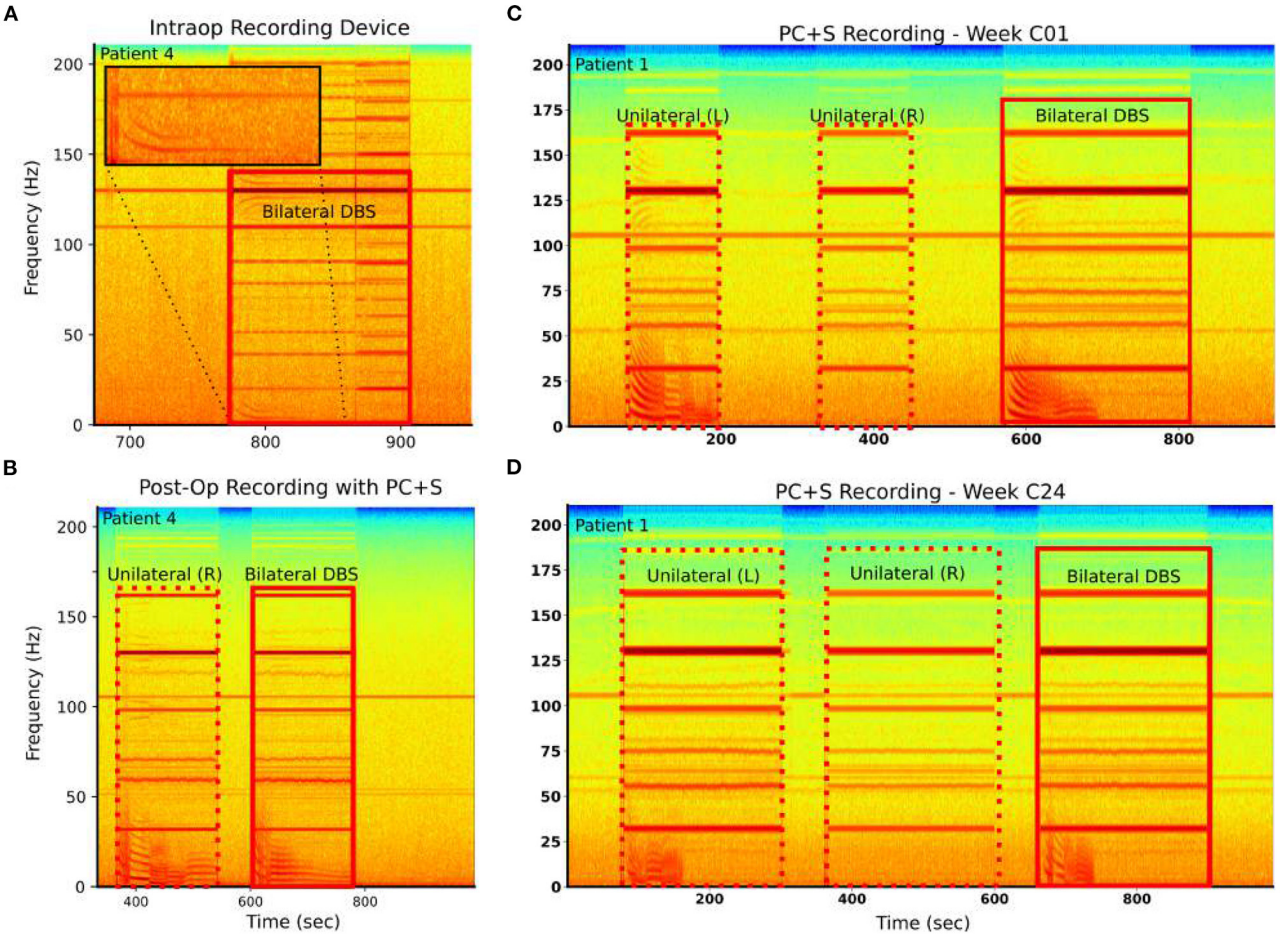
LFP-DOs are robust when present. Example spectograms from raw recordings in two patients: Left column—Patient 4, Right column—Patient 1. **(A)** Patient 4 intraoperative BlackRock Microsystems recordings demonstrated LFP-DOs. **(B)** Patient 4 extraoperative PC+S™ recordings, taken 4 h later demonstrated LFP-DOs with different structure. **(C)** In Patient 1, extraoperative PC+S™ recordings taken during three different stimulation conditions demonstrate DOs in left and bilateral stimulation. **(D)** After 24 weeks of therapy the same experiment still demonstrates DOs evoked by left and bilateral stimulation. However, these DOs exhibit changes in several dynamic features.

DOs were consistently observed for particular combinations of stimulation parameters. LFP-DOs were observed across different data acquisition systems (DAQs) within the same patient (Patient 4) (Figures 4A,B). In both a high-fidelity intraoperative DAQ and the Activa PC+S™ a DO was observed, though differences in the dynamics were evident (Figures 4A,B).

Similarly, LFP-DOs were observed in recordings taken months apart (Figures 4C,D). Here, Unilateral left stimulation was seen to evoke DOs, while unilateral right does not (Figures 4C,D) at both the C01 and C24 weeks. Certain properties of the DO were consistent, like fundamental frequency and harmonics, while other differed, such as reduced total time of DO evolution (Figure 4D).

**Figure 5:**
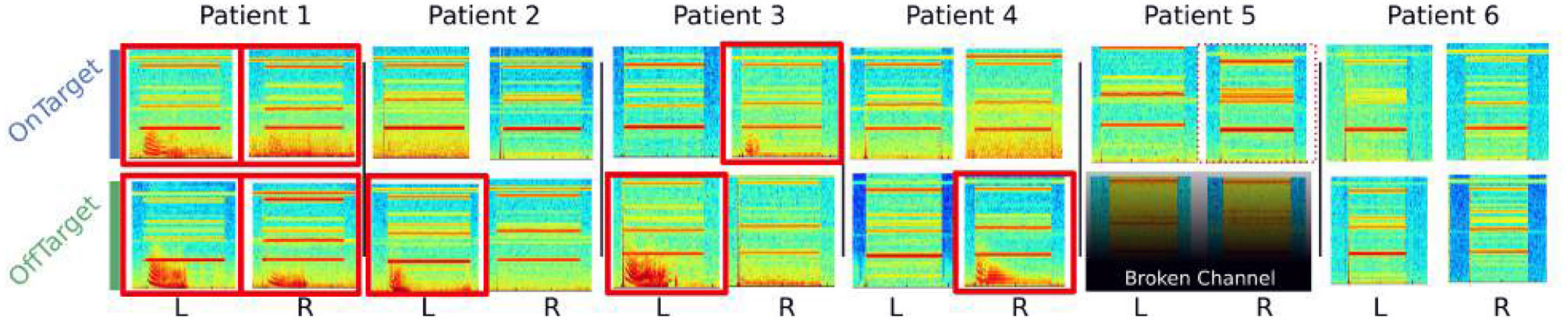
DOs evoked by bilateral stimulation across cohort. Spectrograms from all bilateral stimulation conditions, both OnTarget and OffTarget, in all six patients. Red squares indicate LFP-DO+ conditions. Black blocks indicate recordings involving a broken channel and unsalvagable recordings under OffTarget stimulation.

LFP-DOs were observed in 4/6 patients at different stimulation conditions (Figure 5), mostly in the OffTarget configuration (Figure 5). When present, DOs evolved over the course of minutes, being measured in either left or right SCC-LFP.

### 3.3. DO Characteristics

**Figure 6:**
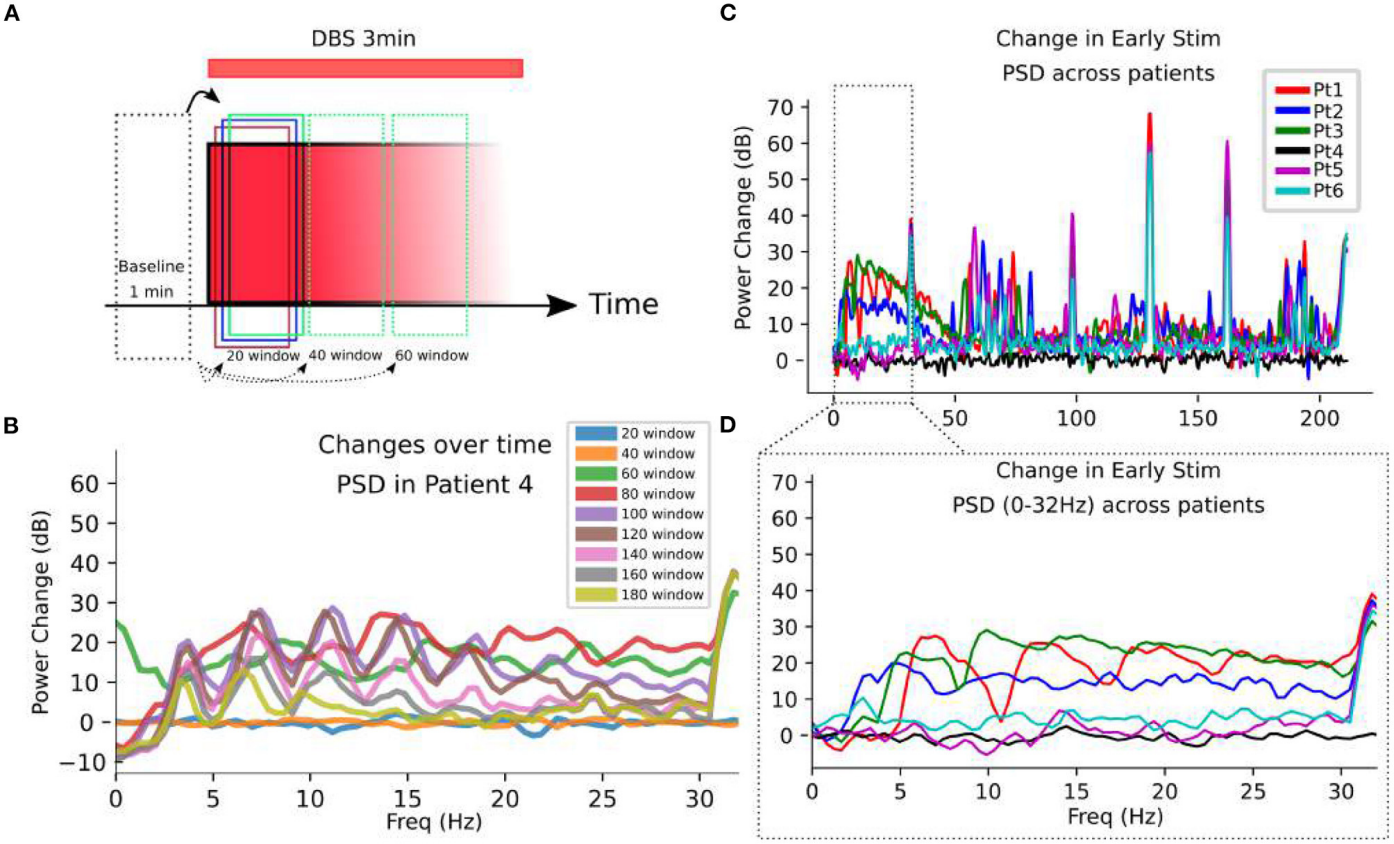
Oscillatory changes evoked by DBS. **(A)** SCC-LFP recordings are taken for 1 min before and 3 min after DBS initiation at a given target. 20 s, non-overlapping windows are analyzed in the frequency domain to observe the changing frequency content over 3 min stimulation. **(B)** Changes in power spectral densities (PSD) are calculated in different 20 s long time windows, with the label referring to the end point of the window. In Patient 4 as an example, the change in oscillatory content is evident as overt changes in the PSD along 20 s non-overlapping windows. **(C)** Log-changes in PSD calculated during the initial 20 s of DBS onset demonstrate significant changes from baseline in a subset of patients. **(D)** A zoom-in to the artifact-free 0 to 30 Hz range demonstrates the frequency-domain activity defining the DO. The PSDs all miss the dynamics that are present over the stimulation period.

Power spectral density (PSD) changes from pre-stimulation baseline is analyzed (Figure 6A). PSD changes from baseline demonstrate DOs are observed only during DBS (Figure 6) Non-stationarity of the PSD is evident in PSD changes along non-overlapping time windows during stimulation (Figure 6). However, this analysis establishes the emergence of significantly large low-frequency power evoked by DBS, with as determined by comparison with stimulation artifacts (Figure 6C).

In all observed DOs, distinct phases of smoothly changing dynamics were observed over the stimulation timecourse, separated by abrupt changes in fundamental frequency and its harmonics (Figure 3D). These abrupt changes in fundamental frequency are visually evident and will be used to define distinct *regimes* within a DO. A summary of properties for these DOs is provided in Table 2 and demonstates the variability of the internal structures of a DO.

**Table 2:** DO regime properties.

**Patient**	**Mean Decay Time**	**Visual Regimes**
Patient 1	125 s	3
Patient 2	100 s	2
Patient 3	112 s	3
Patient 4*	180 s	5
Patient 5	N/A	N/A
Patient 6	N/A	N/A

*Four patients exhibited DOs. The mean decay time, determined by disappearance of the second harmonic of the DO, is characterized for the DO displayed in Figure 5. Visually distinct regimes are determined by distinct discontinuities in the DO fundamental frequency*.

### 3.4. Dynamics of LFP-DOs

To better capture dynamic and non-linear properties of LFP-DOs, we apply the SINDy algorithms to analzse coefficients reflecting canonical types of dynamics.

**Figure 7:**
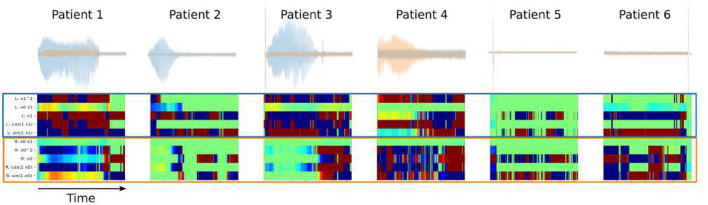
Intrahemispheric Dynamics across LFP-DOs in all patients. First row: measured DOs for each patient, with Left-SCC LFP in blue, Right-SCC LFP in red. Second row: SINDy coefficients for cross-terms - blue block shows coefficients for where Right-SCC LFP affects Left-SCC LFP change, red block shows coefficients for where Left-SCC LFP affects Right-SCC LFP change.

SINDy models trained along a sliding window across the stimulation interval yield cross-coefficient maps (Figure 7) These maps reflect the impact that contralateral LFP has on the dynamics of a given LFP channel. In all DOs, the impact of the right on the left SCC demonstrates large coefficients. The difference in amplitude between the LFP channels may contribute to this asymmetry, but corrections distort the relationships we're attempting to measure. Distinct patches of conserved coefficients are observed across the timecourse of LFP-DOs, suggesting different intervals of DOs are governed by different dynamics.

**Figure 8:**
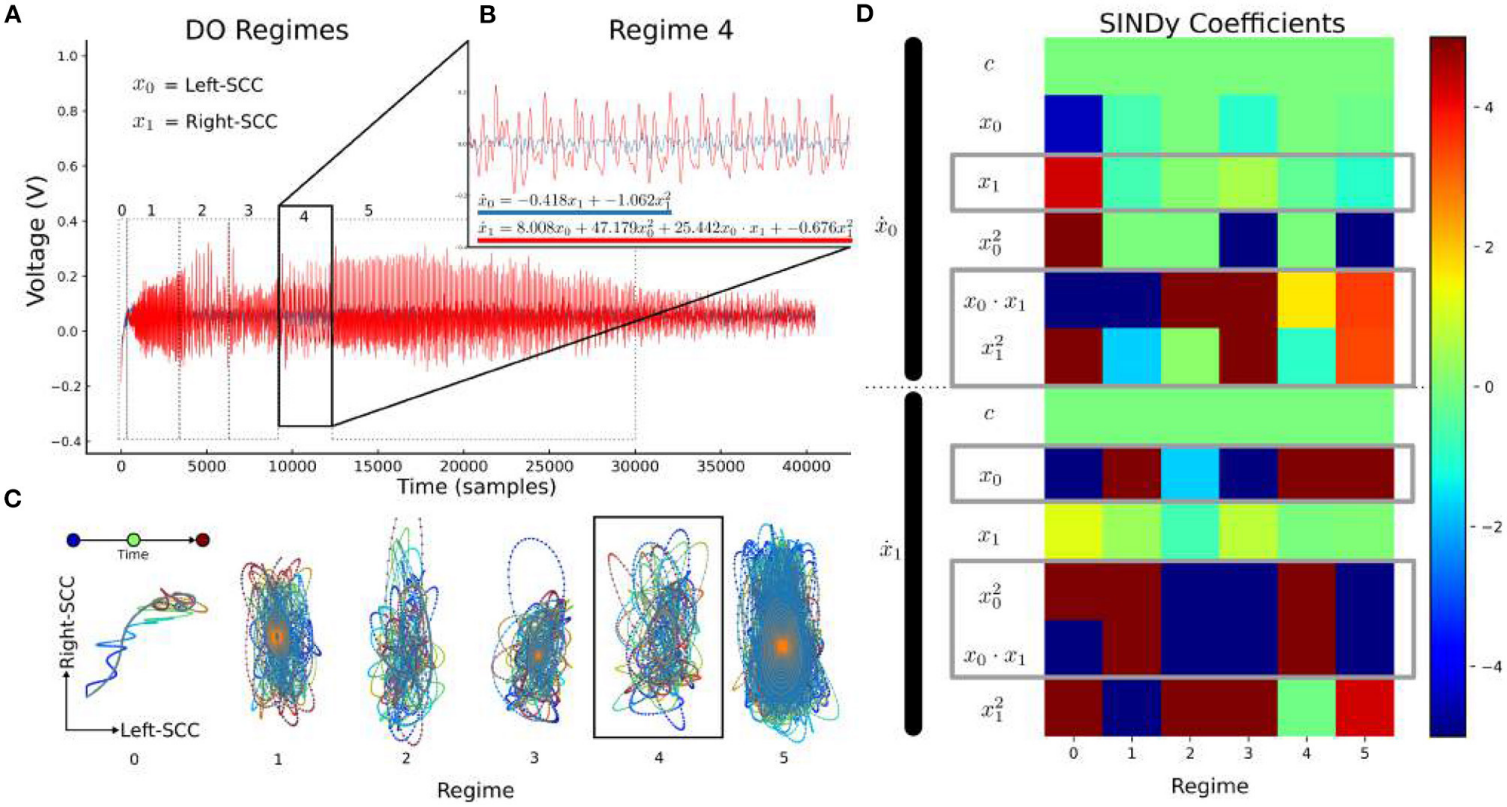
LFP-DO Regime Dynamics in Patient 4 LFP-DO timeseries. Timeseries from Figure 4A is regenerated here with regime markers. **(A)** Distinct regimes are evident in a DO with smoothly changing dynamics. Smoothly changing regimes are interrupted by abrupt changes in fundamental frequency and associated harmonics. **(B)** With the regime-4 as an example, left (blue) and right (red) SCC-LFP recordings appear coordinated. **(C)** Each regime was plotted in phase space with Right-SCC vs. Left-SCC voltages, and color-coded time. **(D)** The regime-4 (black box) empirical trajectory (colors) was used to learn a model (b equation) and identify directional influence between bilateral SCC.

In Patient 4, SINDy analysis is performed on non-overlapping regimes—visually distinct periods of time in the spectrogram (Figure 8A). Here, *x*_0_= left-SCC and *x*_*i*_= right-SCC, and a non-linear dynamics model was learned for each regime (Figure 8C). The resulting dynamics equations for each regime are then compared directly in their coefficients, particularly for “cross-terms:” where the bilateral-SCC influence the other.

### 3.5. Tractography of Observed LFP-DO

**Figure 9:**
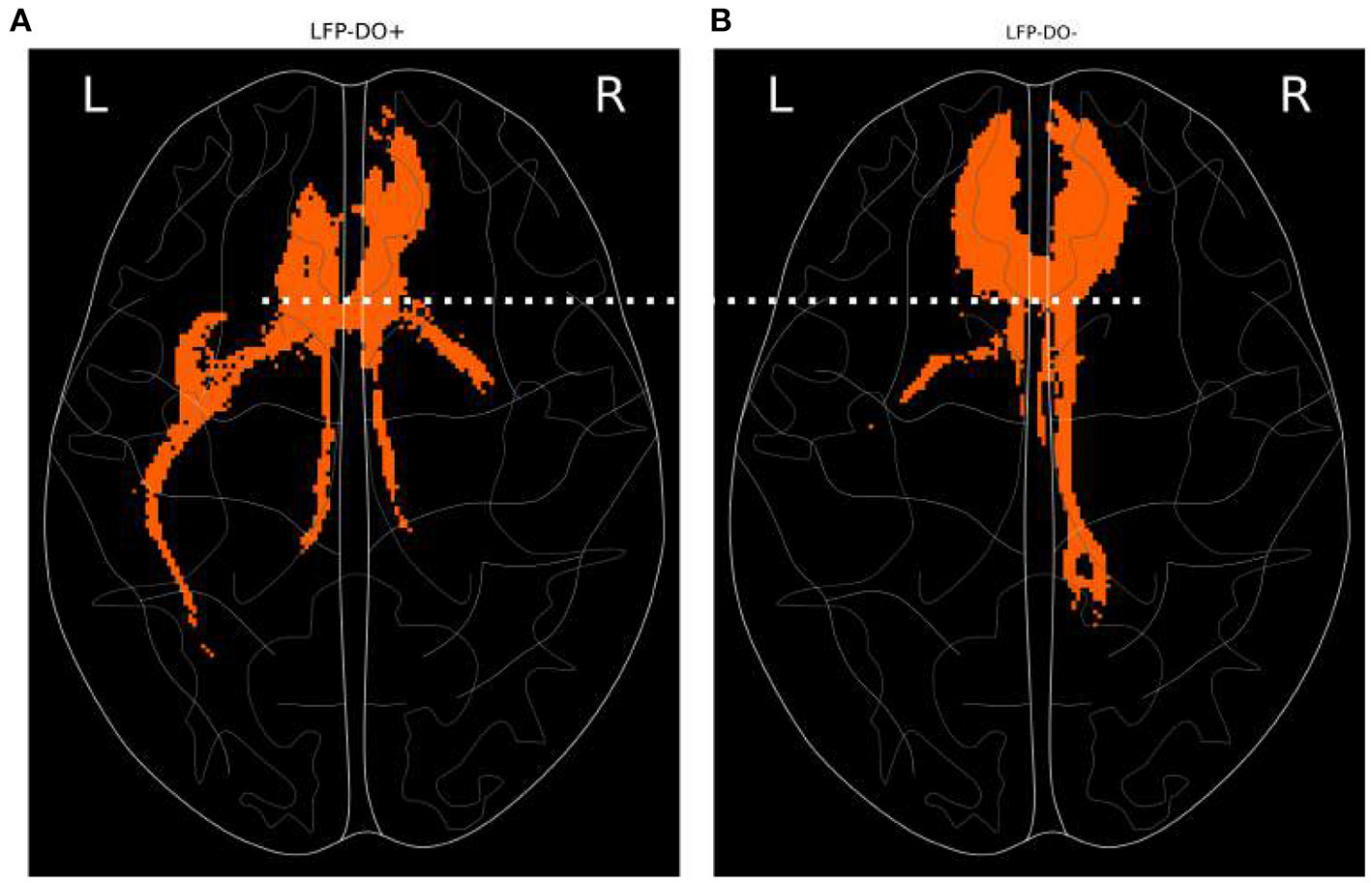
Axial tractography associated with LFP-DO+ across full n=6 patient cohort. Fibers engaged, on average, more with conditions that evoked measurable DOs (LFP-DO+) and conditions that did not evoke measurable DOs (LFP-DO-). Horizontal slice with frontal pole at top. **(A)** LFP-DO+ conditions exhibited more engagement of left-UF while **(B)** LFP-DO- exhibited more engagement of right-CB and Fmin. LFP-DO+ demonstrated more posterior engagement of interhemispheric fibers (white dotted line). UF—Uncinate Fasciculus, CB—Cingulum Bundle, Fmin - Forceps Minor.

*Post-hoc* analysis of stimulated fiber bundles in LFP-DO+ conditions was compared with LFP-DO- conditions across all patients (Figure 9 showing axial view). Tractography identified for each condition is then masked to identify voxels that were, on average, larger in the LFP-DO+ conditions vs. the LFP-DO- conditions (Figure 9A), and the converse (Figure 9B). LFP-DO+ conditions where characterized by more left UF and posterior Fmin (Figure 9A), with bilateral CB engaged symmetrically. In contrast, LFP-DO- conditions were associated with asymmetric engagement of right CB and anterior Fmin bilaterally and evenly (Figure 9). LFP-DO+ tracts were also associated with engagement of more posterior fibers along the midline (Figures 9A,B white-dotted line) while LFP-DO- did not include those voxels.

### 3.6. Remote DO Identification

**Figure 10:**
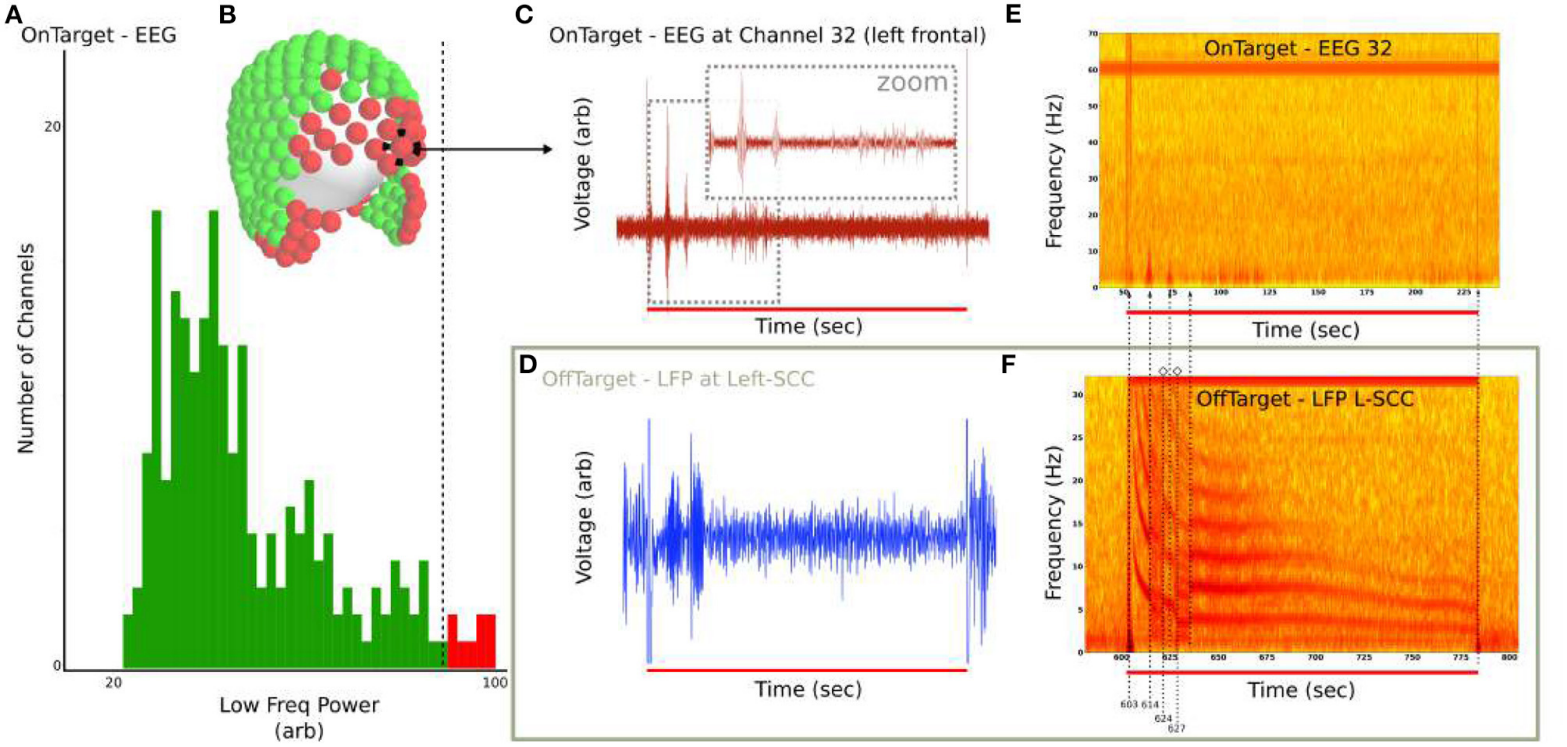
EEG exhibits transient low-frequency activity at DO regime timings in Patient 4 EEG. **(A)** Distribution of maximum 2 to 5 Hz power across all EEG channels. Top 10% of channels were identified and **(B)** marked (red) in sensor space. **(C)** Filtered OnTarget EEG from Channel 32 (frontal) demonstrates bursts at similar timescales as LFP-DO. **(D)** Filtered OffTarget LFP from Left SCC. **(E)** Spectrogram of unfiltered EEG Channel 32 shows 2 to 5 Hz activity. **(F)** Spectrogram of unfiltered LFP L-SCC, aligned with **(E)**, with guides (dotted line) for LFP-DO phases and corresponding hints of DO in EEG.

Under the hypothesis that DOs are also evoked in regions downstream of stimulated tracts, we searched for DOs across 256-channel EEG in the single LFP-DO+ patient with a full LFP and EEG dataset (Patient 4). Filtered 256-channel dEEG is analyzed in the low-frequency 0 to 10 Hz range for (Figure 10A), with max low-frequency power across all time segments calculated for all channels. The top 10% of channels are identified (Figure 10B red channels) and an example frontal channel (Channel 32) shown (left frontal; Figure 10C). The EEG of the OnTarget recording (Figure 10C) was analyzed next to the LFP of the OffTarget recording (Figure 10D) that exhibited an LFP-DO. The time-frequency representations of both stimulation conditions were then aligned to stimulation onset and distinct landmarks of the LFP-DO compared to the putative EEG-DO in EEG channel 32 (Figures 10E,F dotted vertical lines).

## 4. Discussion

Dynamics in LFP have been measured before in epilepsy (Schiff et al., 2000) and DBS (Wiest et al., 2020), reflecting time-varying activity in the brain that can be ignored in traditional analyzes. Here, we observe novel dynamic oscillations (DOs) in local field potentials (LFP) evoked by deep brain stimulation (DBS) within bilateral subcallosal cingulate cortex. Unlike previous reports, the LFP-DOs observed here had low fundamental frequencies, typically between 2 and 20 Hz, evolved over minutes of stimulation (Figure 3D), and were not associated with seizure-like behaviors. These LFP-DOs are associated with stimulation of particular white matter tracts and may serve as a biomarker for target engagement confirmation. Our preliminary characterization of these serendipitous measurements sought to confirm the neural origin of LFP-DOs and to propose.

### 4.1. LFP-DOs Are Neural and Stereotyped

We observed LFP-DOs in four out of six patients, primarily under OffTarget (non-therapeutic) stimulation (Figure 4). The DOs, when present, were present across six months of active therapy with slightly modified characteristics, like shortened evolution time, changes in intra-regime fundamental frequency dynamics, and harmonic structure (Figures 4C,D). DOs are observed in both a high-power intraoperative data acquisition system (Figure 4A, Supplementary Figure 1) as well as the prototype Activa PC+S™ (Figures 4B–D). All patients who exhibited LFP-DOs with the Activa PC+S™ and also had high performance intraoperative LFP recordings (Patients 3 and 4) exhibited DOs in both systems, strengthening the likelihood that LFP-DOs are not device-related artifact. DOs are present only during active DBS, have multiple large peaks in the frequency domain, and the frequencies of these peaks change over the course of seconds and minutes. Significant variability in LFP-DOs exist between patients, suggesting they arise from multiple interacting factors: variable anatomy, variability in stimulated brain circuits, concommitent medications, intrinsic physiology, and differences in lead orientation.

The DO itself exhibited stereotyped signal structure, both in the filtered time domain signal and the time-frequency domain spectrogram views (Figure 3). In the time-domain, DOs appear to have distinct regimes separated by sharp transitions in the activity frequencies present (Figure 3), and further study is needed to understand the significance of distinct regimes and their transitions. Analysing DOs in the frequency and time-frequency domain is challenging due to the non-stationarity and harmonics, which are smeared by the Fourier analysis across the time interval analyzed (Figure 6B, Supplementary Figure 3). For example, any oscillatory analyzes would find time-varying power across all low-frequency bands, implying complex coordinations across distinct oscillations; but we see that a single changing process is likely more parsimonious. Additionally, the dynamics of the DO require imbalanced timeseries lengths to ensure stationarity within an analyzed block, which can introduce bias into estimated PSDs.

### 4.2. DOs Are Dynamic and Non-linear

To better capture the dynamics and non-linearities likely underlying the DOs, we propose the use of SINDy as a complement to traditional analyses. SINDy is a neural-network based algorithm that fits canonical dynamics to multiple timeseries, yielding a sparse set of coefficients for each basis function of a library of non-linear basis functions (Brunton et al., 2016; de Silva et al., 2020). We use SINDy models fit to LFP-DO (Figure 8) to assess dynamics across stimulation periods and compare LFP-DOs across patients (Figure 7). The full SINDy model learned in each patient's DO (including OffTarget stimulation in Patients 5 and 6, who did not have LFP-DOs) demonstrate the inclusion of coefficients for non-linear terms across patients (Supplementary Figure 4), supporting the use of SINDy over traditional linear analyses like Granger Causality. Like Fourier-based analyses, these coefficients are affected by signal amplitude and the LFP channel with a DO consistently exhibit large coefficients (Figure 7, Supplementary Figure 4), however, SINDy provides additional information in the non-linear basis functions, their distinct contributions, and the evolution of those contributions.

Cross-coefficients, coefficients for Left-LFP dynamics involving Right-LFP and *vice versa*, are interpreted as how bilateral SCC affect each other, with directionality (Sugihara et al., 2012; Champion et al., 2019). Consistent across patients is the influence of Right-SCC on Left-SCC (Figure 7 second row, blue box) during DOs, with inconsistent influence of Left-SCC on Right-SCC. Consistent across all LFP-DOs is variability in the sin(*x*_1_) and $x_{1}^{2}$ terms for the Left-SCC dynamics. Coefficients in the Right-SCC dynamics include most of the cross functions, though Right-SCC coefficients are much more likely to be zero at any given moment in time, suggesting Left-SCC does not consistently influence Right-SCC. Significant instability in the coefficients across the stimulation period is evident, suggesting more adaptive windows are needed to ensure stability. With visually determined regimes, analysis of SINDy coefficients within a single LFP-DO demonstrates distinct changes between regimes of a given LFP-DO (Figure 8D), particularly in the asymmetry of influence between Left and Right SCC-LFP DOs.

These results suggest DOs arise from DBS modulation of Left-SCC to Right-SCC dynamics, leaving Right-SCC to Left-SCC dynamics intact to effect downstream changes. The sliding-window SINDy exhibits sustained patterns of coefficients, further development of SINDy in DO analysis can be used to automate regime definitions from full coefficient sets (Supplementary Figure 4). The manual, visual regime analysis supports distinct dynamical regimes being evoked by DBS, potentially secondary to changing neurotransmitter availability following high-frequency depolarization of pre-synaptic terminals in the Right-SCC.

### 4.3. White Matter Associations in LFP-DOs

The region around the SCC is associated with various white matter tracts (Hamani et al., 2011; Riva-Posse et al., 2014; Tsolaki et al., 2021) and stimulation of them is likely to evoke measurable changes in surface measurable cortex. Under the hypothesis that LFP-DOs reflect modulation of brain regions downstream to stimulated white matter, we analyzed dEEG recordings in a single patient that exhibited LFP-DOs (Figure 10). Comparison of LFP-DO+ and LFP-DO- tractography suggests LFP-DOs are associated more with engagement of the left uncinate fasciculus and balanced stimulation of bilateral-CB (Figure 9A). LFP-DO- conditions, largely associated with the therapeutic SCCwm-DBS target, exhibited asymmeric right-CB engagement and symmetric forceps minor, without similar engagement of UF in either hemisphere. Additionally, LFP-DO+ conditions exhibit engagement of most posterior fibers along the midline, potentially reflecting direct SCC-SCC fibers (Figure 9 dotted white line). Engagement of these fibers alone may not necessarily evoke a DO, requiring also engagement of contralateral SCC and UF+CB to stimulate multiple circuits, consistent with the harmonics seen in the LFP-DO and typical of multi-path signals (Schiff et al., 2000; Ridolfi and Win, 2005).

Under the hypothesis that DOs are evoked in brain regions downstream of stimulated white matter tracts, we analyzed dEEG in Patient 4. OnTarget stimulation of SCCwm identified low-frequency activity consistent with DOs, though direct identification is challenging due to the limitations of dEEG. Power in the 2 to 10 Hz range was found primarily in frontal and temporal EEG channels (Figure 10D), consistent with LFP-DO- conditions that demonstrate strong Fmin projections to bilateral frontal regions (Figure 9). The time-alignment of the OnTarget EEG signal and the OffTarget LFP-DO suggests a common underlying generator, despite DBS at two different targets within the SCC in two sessions separated by approximately 30 min (Figure 10F). This analysis is very limited due to the limited availability of dEEG during cohort recruitment and the absence of any LFP-DO in Patients 5-6.

### 4.4. Use as Target Engagement Marker

Identification of neural signals tracking with specific white matter targets is an area of ongoing research and interest, especially as white matter targets for psychiatric illnesses grow (Waters et al., 2018; Segato et al., 2019; Horn and Fox, 2020). Since LFP-DOs are seem mostly in non-therapeutic OffTarget stimulation, they have limited utility as a sensitive signal for target engagement confirmation, but potential DOs in the EEG under OnTarget conditions suggests a role for DOs in assessing network-level engagement in antidepressant SCC-DBS (Waters et al., 2018; Howell et al., 2019, 2020). One potential use of the LFP-DO is as a marker of non-therapeutic engagement within the SCC, informing small adjustments until LFP-DOs vanish.

The harmonic structure of the LFP-DOs are seen in multi-path signal propagation in engineered systems (Schiff et al., 2000; Ridolfi and Win, 2005), suggesting DOs can arise from circuit properties integrated throughout loops, potentially even reflecting multi-synaptic dynamics along canonical circuits like the Circuit of Papez (1937). In that context, the LFP-DO+ tractography is consistent with DOs arising from engagement of a full loop consisting of the uncinate fasciculus and cingulum bundle of the left hemisphere. Notably, the fundamental frequency of 10 Hz corresponds to a time period of 0.10 ms, similar in timescale to traversal of an action potential over the full length of CB (Heilbronner and Haber, 2014; Bubb et al., 2018).

The DO may be a marker of network-level engagement, and we present a preliminary observation of signals in dEEG aligned with DO regimes and exhibiting low-frequency, DO-like behavior (Figure 10). This suggesting DOs can be measured in multiple regions of an engaged network However, more rigorous demonstration in larger cohorts is needed before such a signal in dEEG can be confirmed.

### 4.5. Potential Mechanisms of DOs

Early studies of neural stimulation demonstrated that DBS parameters primarily modulate axons, not gray matter (Nowak and Bullier, 1998; McIntyre et al., 2004), and neurotransmitter release following stimulation can exhibit biphasic responses following depletion of pre-synaptic neurotransmitter (Iremonger et al., 2006). More recent theories build on these studies and propose synaptic suppression as the primary mechanistic effect of DBS (Farokhniaee and McIntyre, 2019) with downstream effects on network-level signaling driving therapeutic responses (McIntyre and Anderson, 2016).

The decay of the fundamental frequency supports neurotransmitter depletion leading to synaptic suppression (Figure 8) over the minutes following stimulation onset (Farokhniaee and McIntyre, 2019). Our observations are consistent with a biphasic neurotransmitter response to DBS (Iremonger et al., 2006; Farokhniaee and McIntyre, 2019), where high-frequency stimulation initially evokes large post-synaptic depolarizations with large-scale, synchronized release of neurotransmitter. The low fundamental frequency in DOs, unlike previous reports in other circuits (Schiff et al., 2000; Wiest et al., 2020), may reflect a physically larger neuronal loops being engaged and modulated, with harmonics reflecting the number of monosynaptic circuits traversed before modulating synaptic inputs into the SCC (Buzsáki et al., 2012; Maling et al., 2018).

Taken together, our observations support the hypothesis that DBS immediately evokes large-scale depolarization and neurotransmitter release, followed by neurotransmitter depletion in pre-synaptic terminal downstream of stimulated white matter tracts. Direct analyzes of DO dynamics are needed to fully extract signals that can test this hypothesis more fully. Further work constraining SINDy to known neural structures, like validated neural mass models, may be necessary to gain more direct insight into the neural components underlying the observed DO (Abbott et al., 2016; Jirsa et al., 2019).

### 4.6. Limitations

There are several major limitations to our findings. First, significant variability is found across patient LFP-DOs, with two patients exhibiting no measurable LFP-DOs (Figure 5). One explanation is that variability in downstream anatomy being stimulated and/or the orientation of the DBS recording lead may explain the variability in LFP-DO structure between patients. Additionally, physiologic factors like concomitant antidepressant use, genetic variability, anatomical variability could all contribute to both the presence and the structure of DOs.

Second, while DOs were observed in multiple recording devices, we have not ruled out all non-neural origins of the DO. In particular, DOs may arise from interactions between the stimulation, recording, and gray-white matter interface. Inter-pulse analysis may be required to definitively verify a neural source for DOs the source of the DO, though the demonstrated non-stationarity of the DO and the PC+S™ limitations preclude “evoked-potential” analyzes at therapeutic stimulation frequencies.

Third, the SINDy algorithm is used here as an analysis tool, not a predictive model. As such, the model fit is not assessed here, though attempts to learn a predictive model of the underlying generative distribution will require cross-validation approaches (Bergmeir and Beńıtez, 2012). Regimes are manually defined with visual inspection of the spectrogram, but a data-driven approach leveraging SINDy goodness-of-fit metrics is needed.

Finally, the preliminary observation of low-frequency activity in dEEG of a single patient is insufficient to confirm a network-level effect. Direct comparison of LFP-DO and EEG signals is complicated by the distance and tissue between neural generators and field potential measurements (Buzsáki et al., 2012; Olson et al., 2016). Larger cohorts are needed to confirm any mechanistic link between LFP-DOs and potential EEG correlates, particularly probing how unilateral stimulations (not used for therapy) contribute to DO properties (Figures 4C,D).

## 5. Conclusion

We report the novel observation of *dynamic oscillations* (DOs) during subcallosal cingulate cortex (SCC) deep brain stimulation (DBS) and perform a preliminary characterization suggesting a network-level mechanism for DO generation. These DOs were observed robustly in local field potentials (LFPs) measured at bilateral SCC, were associated with white matter tract engagement, and exhibited distinct changes in dynamics that appear preserved across large-scale networks. Our preliminary characterization of these DOs can inform broader efforts to identify specific electrophysiologic markers of tractography engagement. Further study of DOs across distributed networks is needed to gain deeper insights into the neural generators of DOs, to build mechanistic models that can inform therapeutic optimization, and to rigorously test DO generation with steerable leads (Timmermann et al., 2015; Panahi et al., 2021).

## Data Availability Statement

The raw data supporting the conclusions of this article will be made available by the authors, without undue reservation. The analysis code in this study can be found in the SCC-DBS Cortical Signatures [github.com/virati/cortical_signatures].

## Ethics Statement

The studies involving human participants were reviewed and approved by Emory University IRB Mount Sinai IRB. The patients/participants provided their written informed consent to participate in this study.

## Author Contributions

VT, KC, RG, and HM experimental design. VT, RG, and HM data acquisition. VT, RB, VJ, and HM analyzes. VT manuscript preparations. All authors provided feedback on drafts and approved the final manuscript.

## Funding

Funding support was provided by the Whitaker International Foundation, National Institutes of Health (UH3NS103550), Hope for Depression Research Foundation and European Union's Horizon 2020 Framework Programme for Research and Innovation under the Specific Grant Agreement No. 945539 (Human Brain Project SGA3). Implanted devices used in the work were donated by Medtronic, Inc. (Minneapolis, MN).

## Conflict of Interest

HM report consulting and intellectual licensing fees from Abbott Labs. RG serves as a consultant to and receives research support from Medtronic, and serves as a consultant to Abbott Labs. The terms of these arrangements have been reviewed and approved by Emory University in accordance with its conflict of interest policies. The remaining authors declare that the research was conducted in the absence of any commercial or financial relationships that could be construed as a potential conflict of interest.

## Publisher's Note

All claims expressed in this article are solely those of the authors and do not necessarily represent those of their affiliated organizations, or those of the publisher, the editors and the reviewers. Any product that may be evaluated in this article, or claim that may be made by its manufacturer, is not guaranteed or endorsed by the publisher.

## Supplementary Material

Supplementary Figure 1 Intraoperative Recordings Confirm LFP-DOs. Four patients had intraoperative recordings with a high-performance data acquisition system. Two of those four patients exhibited LFP-DOs when measuring with the PC+S and with the intraoperative system: Patient 3 and Patient 4. Each row is simultaneous recordings from two different LFP channels in the same experiment. These experiments are from a different protocol and are not further analyzed in this report.

Supplementary Figure 2 Baseline Power before LFP-DOs. Baseline PSD for channels with DOs plotted for each patient.

Supplementary Figure 3 Evolution of PSD following DBS onset. Plots of the change from baseline in each patient demonstrates significant dynamics across the 3 min of stimulation in patients with DOs (Patients 1–4).

Supplementary Figure 4 Full SINDy Coefficients for DOs in all Patients. Coefficients from combined Polynomial and Fourier feature libraries from sliding-window analysis of DOs.
