# Supplementary material for: Dynamic Oscillations Evoked by Subcallosal Cingulate Deep Brain Stimulation
Source: Front Neurosci. 2022 Feb 23;16:768355. doi: 10.3389/fnins.2022.768355 (PMC8905359; doi:10.3389/fnins.2022.768355)
Supplement: Supplementary Figure 1 — Intraoperative Recordings Confirm LFP-DOs. Four patients had intraoperative recordings with a high-performance data acquisition system. Two of those four patients exhibited LFP-DOs when measuring with the PC+S and with the intraoperative system: Patient 3 and Patient 4. Each row is simultaneous recordings from two different LFP channels in the same experiment. These experiments are from a different protocol and are not further analyzed in this report. [file Data_Sheet_1.pdf]

Supplementary Material

1 SUPPLEMENTARY TABLES AND FIGURES

1.1 Figures

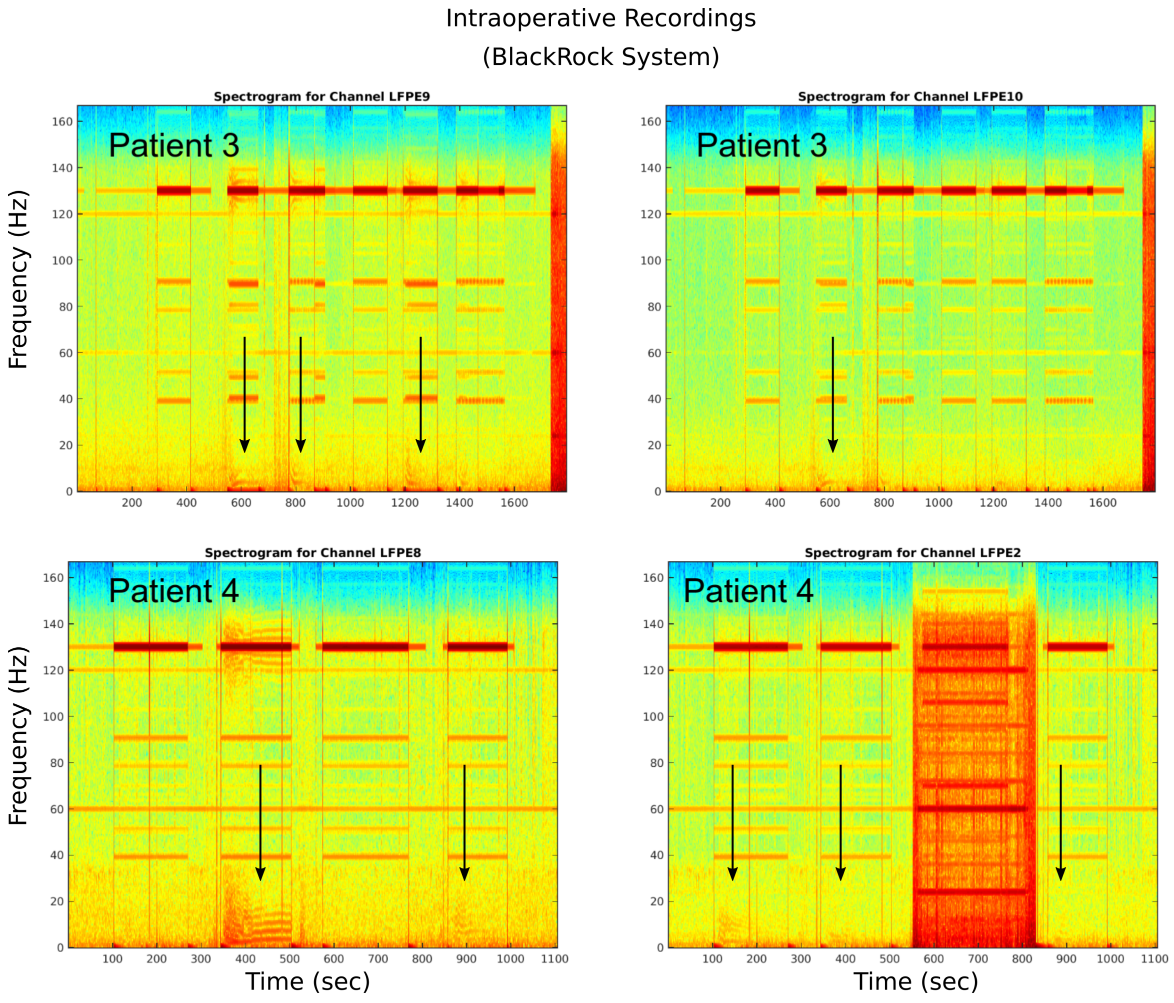

Figure S1. Intraoperative Recordings Confirm LFP-DOs. Four patients had intraoperative recordings with a high-performance data acquisition system. Two of those four patients exhibited LFP-DOs when measuring with the PC+S and with the intraoperative system: Patient 3 and Patient 4. Each row is simultaneous recordings from two different LFP channels in the same experiment. These experiments are from a different protocol and are not further analysed in this report.

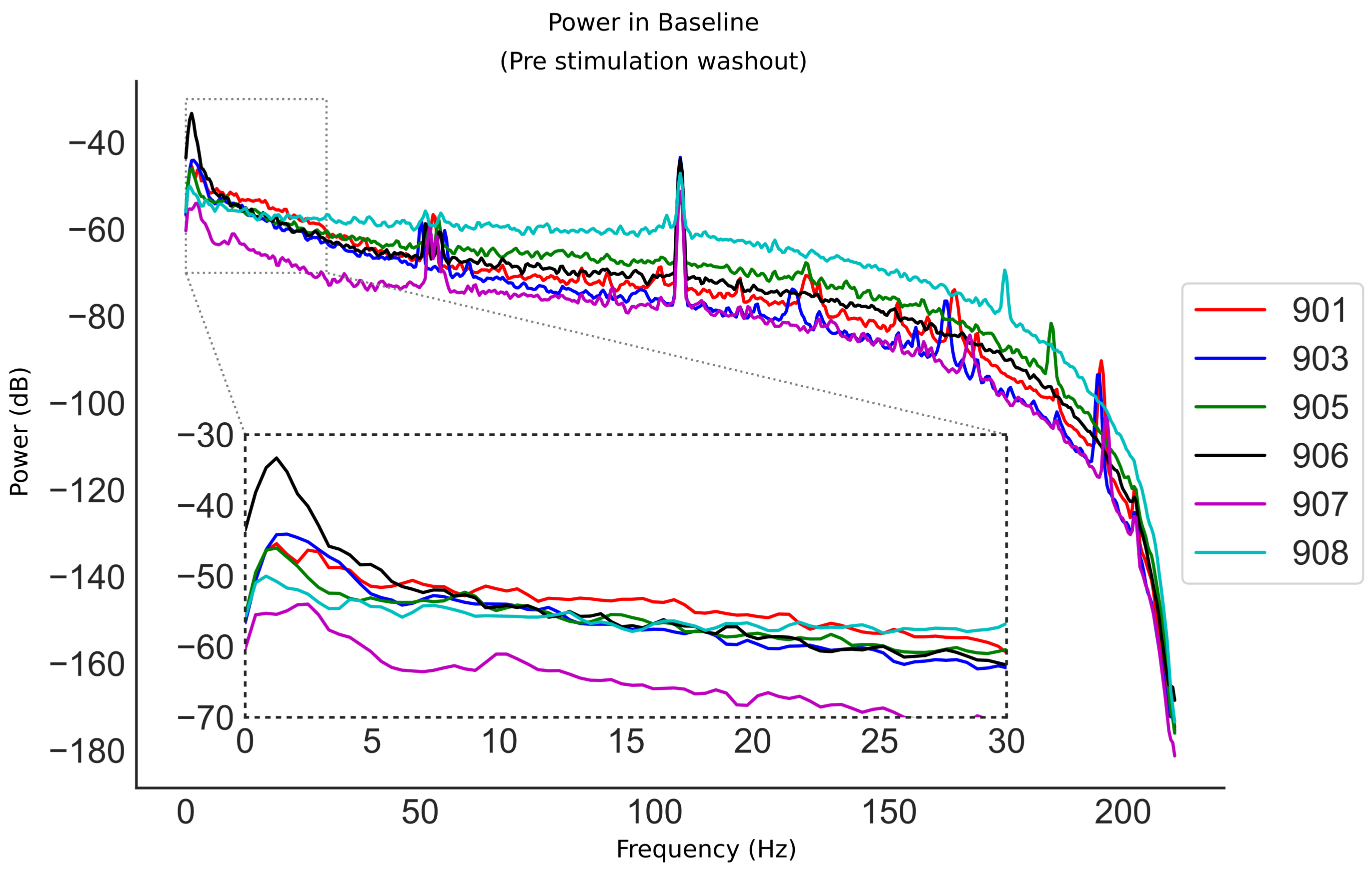

Figure S2. Baseline Power before LFP-DOs. Baseline PSD for channels with DOs plotted for each patient.

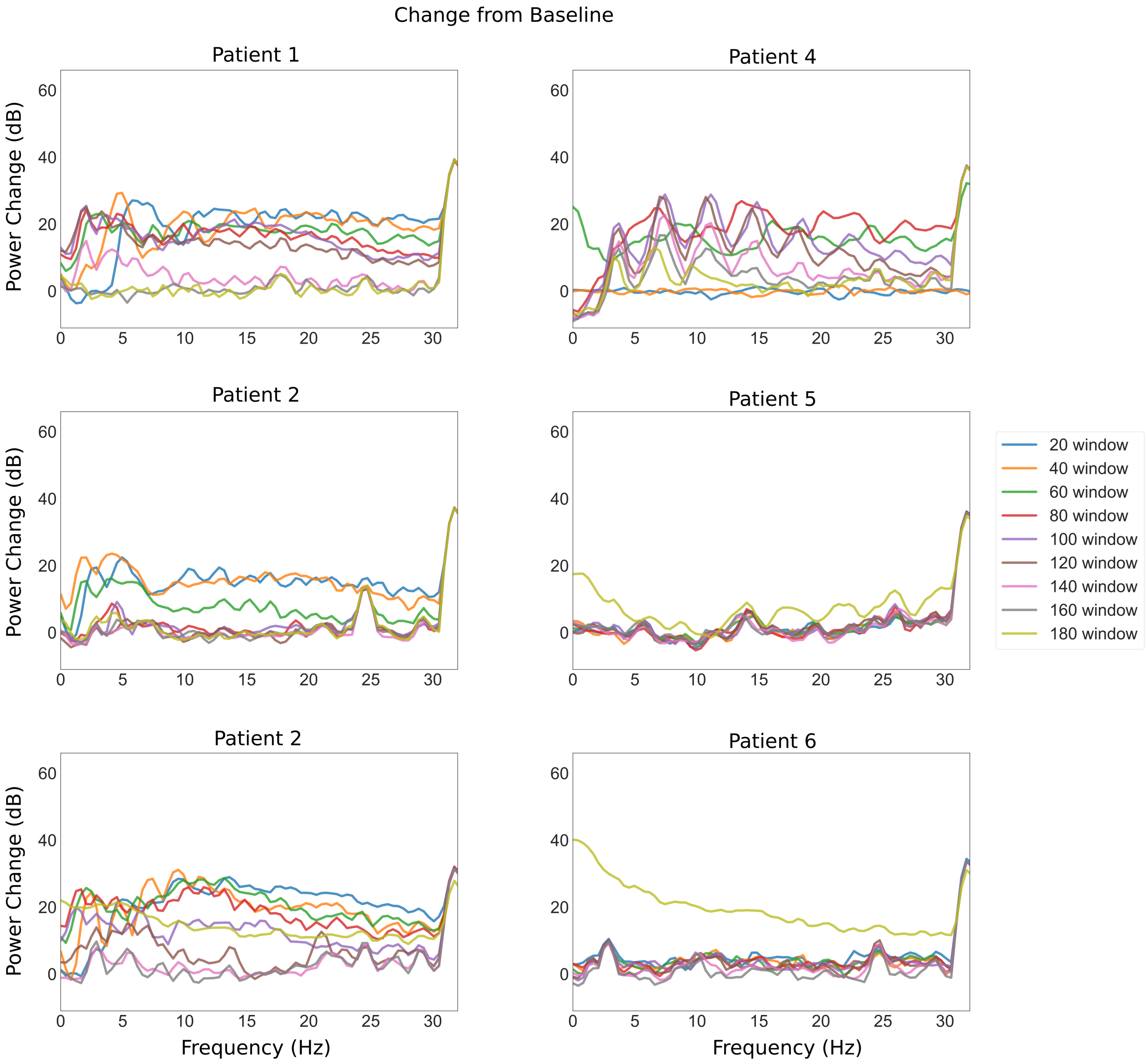

Figure S3. Evolution of PSD following DBS onset. Plots of the change from baseline in each patient demonstrates significant dynamics across the three minutes of stimulation in patients with DOs (Patients 1-4).

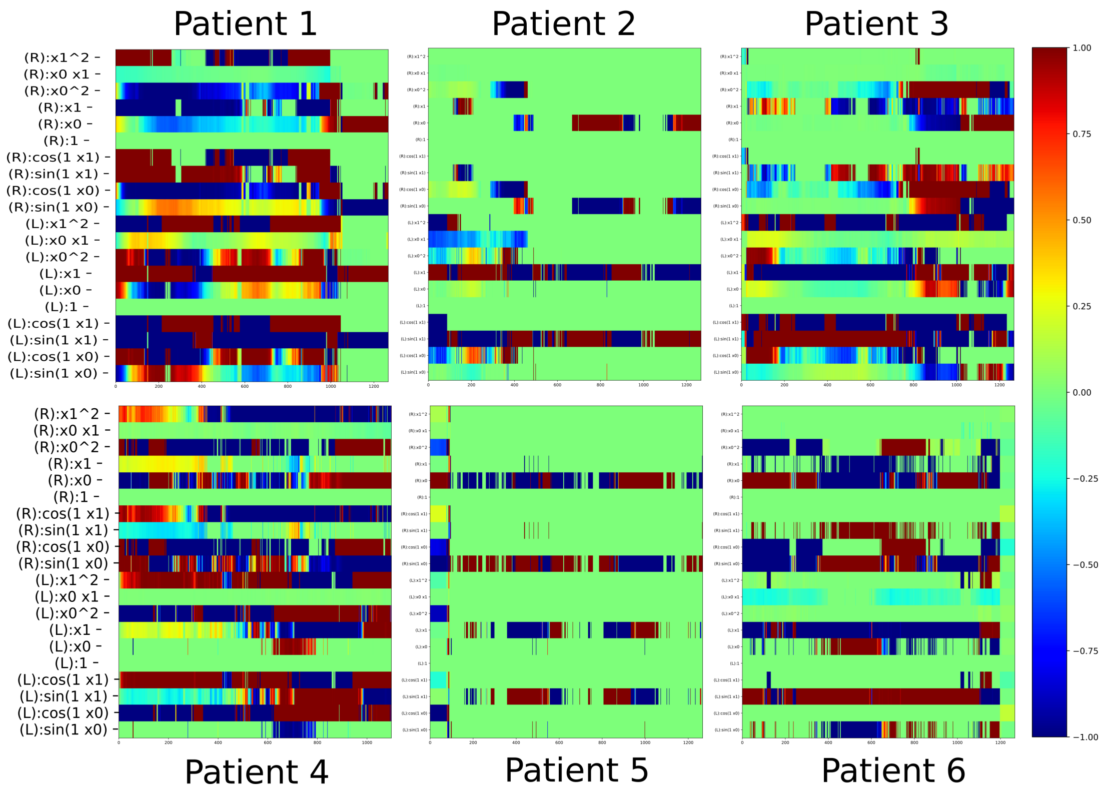

Figure S4. Full SINDy Coefficients for DOs in all Patients. Coefficients from combined Polynomial and Fourier feature libraries from sliding-window analysis of DOs.
